# Adaptive fractional-order non-singular terminal sliding mode control for omnidirectional quadrotors based on WRBF neural network

**DOI:** 10.1371/journal.pone.0339493

**Published:** 2025-12-26

**Authors:** Rui Ma, Qiang Gu, Li Ding, Yuan Li, Changyan Sun, Hongtao Wu

**Affiliations:** 1 Jiangsu University of Technology, Changzhou, China; 2 Zhifang Engineering Design Co., Ltd., Nanjing, China; 3 Nanjing University of Aeronautics and Astronautics, Nanjing, China; Beijing University of Posts and Telecommunications, CHINA

## Abstract

This paper presents a novel robust six-degree-of-freedom trajectory tracking control strategy for tilt-rotor quadrotors operating under uncertainties and disturbances. The key contribution lies in a unified framework that synergistically co-designs a Fractional-Order Nonsingular Terminal Sliding Mode Controller (FONTSMC) with an adaptive Wavelet Radial Basis Function (WRBF) neural network, establishing a deeply integrated architecture rather than a simple combination of independent modules. This co-designed structure introduces three fundamental advances: first, the WRBF network enables precise online estimation and compensation of unstructured uncertainties while the fractional-order nonsingular terminal sliding surface ensures fast finite-time convergence without singularity; second, the Mexican Hat wavelet activation function significantly enhances local approximation accuracy, learning speed, and noise robustness compared to conventional Gaussian RBF networks; third, a parallel control structure integrated with Moore-Penrose pseudo-inverse-based allocation efficiently maps synthesized 6-DOF commands to redundant actuators. Closed-loop stability is rigorously guaranteed through Lyapunov analysis. Comprehensive simulations demonstrate that the proposed controller outperforms conventional NTSMC and RBF-FONTSMC methods in tracking accuracy, convergence speed, control effort, and response smoothness, confirming its superior capability for complex UAV operations.

## Introduction

Recently, drones are widely utilized across various fields owing to their maneuverability, flexibility, and advanced control capabilities. Typical applications include power line inspection, firefighting, land surveying, and precision agriculture [1–4]. As research advances, various forms of UAVs with reliable and enhanced performance have been developed, including multi-rotor [5,6], fixed-wing [7] and hybrid UAVs [8]. However, traditional multi-rotor drones are underactuated, and the inherent coupling between position and attitude hinders six-degree-of-freedom omnidirectional motion, thus limiting their applicability in specialized tasks. To overcome these limitations, the concept of an independently tilting quadrotor has been introduced [9]. The tilting-rotor mechanism converts the multi-rotor UAV from an underactuated to an overactuated system, effectively decoupling position and attitude dynamics [10]. Nevertheless, the unique dynamics of the tilting mechanism, combined with the Coriolis effect and external disturbances, significantly complicate controller design [11].

To address these challenges, various advanced control strategies have been explored, including hybrid control [12], model predictive control (MPC) [13], adaptive control [14,15], finite-time control [16], backstepping control [17] and sliding mode control (SMC) [18]. Among these, SMC has attracted continuous attention due to its simplicity, ease of tuning, and inherent robustness to matched uncertainties [19]. As pointed out in a recent studies, the selection of an appropriate sliding surface and the associated control gain plays a decisive role in determining the overall performance of SMC [20]. However, traditional SMC ensures only that the system state gradually approaches the equilibrium point over time, without guaranteeing finite-time convergence [21,22]. The introduction of Non-singular Terminal SMC (NTSMC) effectively addressed this issue, guaranteeing finite-time convergence while avoiding singularity problems in the control law [23–25]. The versatility and robustness of NTSMC have been demonstrated across diverse systems. These include achieving continuous finite-time control in servo motors [26], stabilizing underactuated flexible joint robots [27], and implementing practical adaptive fast terminal sliding mode control [28]. The fast nonsingular terminal sliding mode control with angular velocity planning presented in [29] provides a novel and effective solution for the full attitude control of quadrotors, demonstrating improved convergence performance.

Nevertheless, under significant system noise, sliding mode controllers may still experience chattering, which can affect control accuracy and actuator lifespan [30]. To alleviate this limitation, Fractional-Order Sliding Mode Control (FONTSMC) was introduced by incorporating the memory and hereditary characteristics of fractional calculus, which helps smooth control action while retaining robustness [31–33]. This approach not only effectively suppresses chattering but also demonstrates enhanced capability in handling complex nonlinear dynamics [34], as evidenced by its successful applications in multi-joint robots [35] and servo systems [36]. More recent studies have reported fixed-time FONTSM schemes for permanent magnet synchronous motors, showing faster convergence characteristics and improved disturbance rejection [37], as well as extreme learning machine-enhanced super-twisting controllers that maintain smooth operation under system uncertainties [38]. Although Fractional-Order Nonsingular Terminal SMC (FONTSMC) integrates finite-time convergence with reduced chattering characteristics [39–41], its performance remains strongly dependent on the availability of accurate system models, which are exceedingly difficult to obtain for highly coupled, nonlinear systems like tilt-rotor UAVs [42].

To alleviate the dependency on precise modeling, various robust compensation techniques have been proposed [43–45]. Among these, neural networks (NNs) stand out due to their powerful approximation capabilities and online learning potential [46]. For instance, Radial Basis Function (RBF) NNs have been successfully employed to estimate disturbances for quadrotors [47]. Furthermore, the robustness of RBFNNs has been extended to fault-tolerant control, where they are utilized for online approximation of system faults and uncertainties in UAV systems, enhancing reliability under actuator failures [48]. However, the performance bottlenecks of conventional Gaussian RBFs—namely, their limited generalization and slow convergence under highly nonlinear dynamics—are increasingly recognized. This limitation was evident in our previous FONTSMC work for a cable-driven manipulator, where the traditional RBF network ultimately constrained performance in dynamic scenarios [49].

To overcome these challenges, Wavelet Radial Basis Function (WRBF) neural networks are considered as a alternative. The Mexican Hat wavelet activation function provides enhanced local approximation accuracy, inherent noise robustness, and faster convergence. The effectiveness of WRBF has been reported in a number of existing studies, including applications in simplified sliding mode control for chattering reduction [50] and GA-optimized precise tracking for robotic systems [51]. Notably, in the complex task of synchronizing fractional-order chaotic systems under time-varying perturbations, WRBF networks have demonstrated superior capability in handling intricate nonlinear dynamics compared to conventional approaches. [52] These demonstrated advantages motivate the integration of the advanced WRBFNN into the present work to handle the complex uncertainties of tilt-rotor flight.

Despite these notable advancements, a fundamental limitation persists: most existing works either focus on improving the sliding surface itself or treat the intelligent compensator as an independent, add-on module in a "simple combination" with the controller. This paradigm leads to three core shortcomings:

1. Loose Integration: The controller and observer are often designed separately, lacking cohesive stability analysis based on a unified Lyapunov function and synergistic adaptive laws. This separation weakens the theoretical guarantee of overall system stability and consistency.
2. Platform Mismatch: Existing schemes are primarily tailored for relatively simpler systems like conventional underactuated quadrotors or servo motors. They fail to fully account for the unique challenges of the over-actuated tilt-rotor platform, namely its strongly coupled 6-DOF dynamics and the critical need for actuator redundancy management.
3. A Comprehensive Solution for a Complex Platform: The proposed framework presents a unified solution through the co-design of a parallel 6-DOF control strategy and a pseudo-inverse-based allocation module, which provides a direct link between control law computation and the corresponding actuator command mapping for the fully actuated platform.

Consequently, this paper proposes a co-designed control architecture for tilt-rotor UAVs, rather than a simple combination of individual methods. The main contribution is the deep integration of FONTSMC with a WRBFNN, which creates a synergistic control mechanism: the precise, real-time disturbance estimation provided by the WRBFNN’s wavelet basis actively reduces the uncertainty burden on the controller, thereby allowing the finite-time convergence and inherent smoothing properties of the fractional-order sliding mode to operate with maximal efficiency and minimal chattering. As a result, the proposed scheme provides a practical means of applying advanced robust control to complex over-actuated platforms.

The novelty and contributions of this work are summarized as follows:

1. A Novel, Deeply Integrated WRBF-FONTSMC Architecture: The adaptive WRBF neural network and the FONTSMC law are co-designed. This creates a synergistic loop: the WRBFNN’s real-time and precise disturbance estimation actively reduces the uncertainty burden on the FONTSMC, allowing its fractional-order dynamics to operate with maximal efficiency and minimal chattering. This is supported by a unified Lyapunov-based stability analysis and tailored adaptive laws, ensuring theoretical coherence and guaranteed performance.
2. Performance Enhancement via Wavelet Functions: The adoption of the Mexican Hat wavelet function as the NN activation unit significantly improves local approximation capability and learning speed, leading to more accurate estimation of the complex lumped uncertainties encountered in tilt-rotor flight.
3. Novel Application to Over-Actuated System Control: The integration of FONTSMC and WRBF for application to complex over-actuated tilt-rotor UAV platforms to achieve decoupled six-degree-of-freedom trajectory tracking represents a novel contribution. This research advances beyond joint-space control or simpler systems by addressing the unique challenges of actuator redundancy and strongly coupled dynamics through a parallel control structure combined with a pseudo-inverse-based control allocation module.

Through this synergistic design, the proposed controller inherits the benefits of finite-time convergence and low chattering from FONTSMC, while simultaneously overcoming the dependency on accurate models via the dynamic learning capability of the WRBFNN, ultimately achieving high-precision and robust 6-DOF trajectory tracking in the presence of uncertainties and disturbances.

The remainder of this paper is organized as follows. The Modeling section presents the system modeling of the tilt-rotor quadrotor UAV. The Controller design section details the design of the proposed WRBF-enhanced FONTSM controller and its stability analysis. Subsequently, Simulation and result analysis section provides comparative simulation results to validate the superiority of the proposed method. Finally, the paper is concluded in the Conclusion section, and potential future research directions are discussed in the Discussion section.

**Remark 1.** The main contribution of this work, relative to existing methods, lies in the integrated co-design of FONTSMC and WRBFNN into a unified architecture. Unlike existing approaches that treat these components as independent or simply cascaded modules, our deep integration establishes a closed-loop synergy: the WRBFNN’s real-time precise estimation actively reduces the uncertainty burden on the FONTSMC, enabling it to operate with maximal efficiency and minimal chattering. This novel architecture is specifically designed to solve the core challenges of strongly coupled dynamics and actuator redundancy in over-actuated tilt-rotor UAVs, demonstrating significant advantages over conventional control strategies.

## Modeling

### Coordinates and orientation

**Fig 1:**
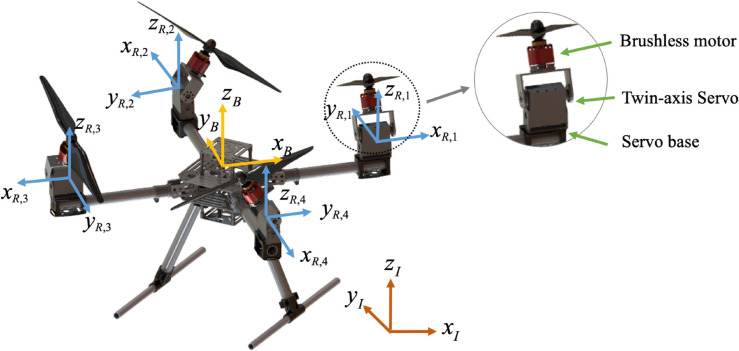
Coordinate system for tiltable quadrotor.

To describe the spatial configuration and dynamic behavior of the tiltable quadrotor system, three coordinate frames are defined. The inertial frame ${ℱ}_{I}=\{O_{I}\;X_{I}\;Y_{I}\;Z_{I}\}$ is fixed to the ground and used to present the global position and orientation of the UAV. The body frame ${ℱ}_{B}=\{O_{B}\;X_{B}\;Y_{B}\;Z_{B}\}$ is attached to the center of mass of the vehicle and moves with it. Each rotor is assigned a local rotor frame ${ℱ}_{R,i}=\{O_{R,i}\;X_{R,i}\;Y_{R,i}\;Z_{R,i}\}$ where *X*_*R*,*i*_ points outward along the arm and *Z*_*R*,*i*_ is aligns with the thrust direction of the rotor. The quadrotor arms are symmetrically distributed in a cross configuration, and the rotors are numbered counterclockwise starting from the front-left rotor *R*_1_, as illustrated in Fig 1.

The orientation of the UAV with respect to the inertial frame is described using the ZYX Euler angle sequence, denoted as $\Theta =[\phi ,\theta ,\psi {]}^{T}$, where ϕ, θ and ψ correspond to the roll, pitch and yaw angles, respectively. The rotation matrix from the body-fixed frame to the inertial frame is defined as:

$$R_{B}^{I}=[\begin{array}{ccc} \cos\theta \cos\psi & \sin\phi \sin\theta \cos\psi -\cos\phi \sin\psi & \cos\phi \sin\theta \cos\psi +\sin\phi \sin\psi \\ \cos\theta \sin\psi & \sin\phi \sin\theta \sin\psi +\cos\phi \cos\psi & \cos\phi \sin\theta \sin\psi -\sin\phi \cos\psi \\ -\sin\theta & \sin\phi \cos\theta & \cos\phi \cos\theta \end{array}]$$ (1)

The orientation of ${ℱ}_{R,i}$ with respect to ${ℱ}_{B}$ is denoted as:

$$R_{R,i}^{B}=R_{z}\left((1-i)\frac{\pi }{2}\right),\;i=1,\cdots ,4.$$ (2)

where the rotation matrix about the z-axis is defined as:

$$R_{z}(\theta )=[\begin{array}{ccc} \cos\theta & \sin\theta & 0\\ -\sin\theta & \cos\theta & 0\\ 0 & 0 & 1 \end{array}]$$ (3)

The position vector at the origin of ${ℱ}_{R,i}$ in the body frame ${ℱ}_{B}$ is

$$p_{R,i}^{B}=R_{R,i}^{B}{\left[\begin{array}{ccc} l & 0 & 0 \end{array}\right]}^{T},\;i=1,\ldots ,4.$$ (4)

where *l* is length of the quadrotor arm.

### Rigid body model

To describe the motion characteristics of the tiltable quadrotor UAV, a six degree of freedom (6-DOF) rigid body dynamics model is established using the Newton-Euler formalism. It is assumed that the UAV performs unconstrained motion, with its position P and orientation 𝛩 defined in the inertial ${ℱ}_{I}$ frame as follows:

$$\left\{ \begin{array}{c} \dot{P}=V\\ \dot{𝛩}=W(𝛩)\;\omega \end{array}\right.$$ (5)

where $𝛩={\left[\begin{array}{ccc} \phi & \theta & \psi \end{array}\right]}^{T}$ represents the Euler angles; $P={\left[\begin{array}{ccc} x & y & z \end{array}\right]}^{T}$ is the position vector; $V={\left[\begin{array}{ccc} u & v & w \end{array}\right]}^{T}$ is the velocity vector in the inertial frame; $\omega ={\left[\begin{array}{ccc} p & q & r \end{array}\right]}^{T}$ denotes the angular velocity expressed in the body frame; The matrix W(𝛩) satisfies:

$$W(\Theta )=[\begin{array}{ccc} 1 & \tan\theta \sin\phi & \tan\theta \cos\phi \\ 0 & \cos\phi & -\sin\phi \\ 0 & \frac{\sin\phi }{\cos\theta } & \frac{\cos\phi }{\cos\theta } \end{array}]$$ (6)

The translation dynamics of the UAV in the inertial frame ${ℱ}_{I}$ are described by:

$$m\ddot{P}=mge_{3}+R_{B}^{I}F_{p}+F_{d}$$ (7)

where $e_{3}=[0,0,1{]}^{T}$; $F_{p}$ is the total thrust vector generated in the body frame; $F_{d}$ denotes the lumped disturbance force, which includes modeling errors and unknown external disturbances.

Then rotation dynamics described in ${ℱ}_{B}$ are:

$$J_{b}\dot{\omega }=-\omega \times J_{b}\omega +M_{p}+M_{d}$$ (8)

where $J_{b}$ is the inertia matrix of the quadrotor; $M_{p}$ is the control moment generated by the rotors; $M_{d}$ stands for external disturbances and unmodeled effects.

The above equations fully describe the translational and rotational dynamics of the tilt-rotor quadrotor UAV.

### Aerodynamics

**Fig 2:**
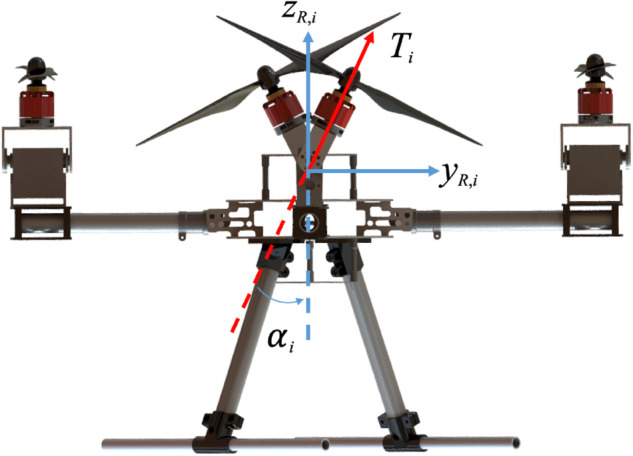
Coordinate system for tiltable quadrotor.

The aerodynamic model of the tiltable quadrotor establishes the mapping between each rotor’s variables and the corresponding aerodynamic forces and moments applied to the UAV body. The coordinate frames and reference axes involved in the modeling process are illustrated in Fig 2.

Let the angular velocity and tilting angle of the *i*-th rotor be denoted as *n*_*i*_ and $\alpha_{i}$, respectively. Assuming that the generated thrust and torque are proportional to the square of the rotor speed, the thrust vector $T_{i}$ and the counteracting torque $M_{i}$ in the local rotor frame ${ℱ}_{R,i}$ are defined as:


$$T_{i}={\left[\begin{array}{ccc} 0 & -k_{t}n_{i}^{2}\sin(\alpha_{i}) & k_{t}n_{i}^{2}\cos(\alpha_{i}) \end{array}\right]}^{T}$$

$$M_{i}={\left[\begin{array}{ccc} 0 & \left(-1{)}^{i}c_{d}k_{t}n_{i}^{2}\sin(\alpha_{i}\right) & \left(-1{)}^{i}c_{d}k_{t}n_{i}^{2}\cos(\alpha_{i}\right) \end{array}\right]}^{T}$$ (9)

where *k*_*t*_ is rotor thrust coefficient; *c*_*d*_ is the lift-to-drag ratio; $\alpha_{i}$ is the active tilting joint angle of the *i*-th rotor about its arm axis *x*_*R*,*i*_ .

To obtain the resultant aerodynamic force $F_{p}$ and moment $M_{p}$ acting at the UAV’s center of mass in the body frame ${ℱ}_{B}$, all individual forces and moments are transformed from their respective rotor frames via the corresponding rotation matrices $R_{R,i}^{B}$:

$$F_{p}=\sum_{i=1}^{4}R_{R,i}^{B}T_{i}$$ (10)

$$M_{p}=\sum_{i=1}^{4}\left(R_{R,i}^{B}M_{i}+p_{R,i}^{B}\times R_{R,i}^{B}T_{i}\right)$$ (11)

Combining the above expressions, the overall aerodynamic model can be compactly represented as a linear mapping between a generalized thrust vector and the combined wrench. Therefore, this model can be written as:

$$[\begin{array}{c} F_{p}\\ M_{p} \end{array}]=A𝛺$$ (12)

where A is a static allocation matrix, as shown in (13), that only depends on the geometry of the quadrotor. 𝛺 is defined as the vector of all the components generated by the individual rotors, as shown in (14).

$$A=[\begin{array}{cccccccc} 0 & 0 & -1 & 0 & 0 & 0 & 1 & 0\\ 1 & 0 & 0 & 0 & -1 & 0 & 0 & 0\\ 0 & 1 & 0 & 1 & 0 & 1 & 0 & 1\\ 0 & 0 & 0 & L & 0 & 0 & 0 & -L\\ -c & -L & 0 & 0 & c & L & 0 & 0\\ L & c & L & -c & L & c & L & -c \end{array}]$$ (13)

$$𝛺=[\begin{array}{c} -k_{t}n_{1}^{2}\sin(\alpha_{1})\\ k_{t}n_{1}^{2}\cos(\alpha_{1})\\ \vdots \\ -k_{t}n_{4}^{2}\sin(\alpha_{4})\\ k_{t}n_{4}^{2}\cos(\alpha_{4}) \end{array}]$$ (14)

### Problem formulation

To simplify subsequent controller design, the complete translational and rotational dynamics of the tiltable quadrotor UAV, as derived in Sections Rigid body model and Aerodynamics, are reformulated into a unified second-order nonlinear system:

$$\ddot{x}=C+Bu+D$$ (15)

with,

$$C=[\begin{array}{c} G\\ J_{b}^{-1}\left(-\omega \times (J_{b}\omega )\right) \end{array}],\;B=[\begin{array}{cc} \frac{R_{B}^{I}}{m} & 0_{3\times 3}\\ 0_{3\times 3} & J_{b}^{-1} \end{array}],\;D=[\begin{array}{c} \frac{F_{d}}{m}\\ J_{b}^{-1}M_{d} \end{array}]$$ (16)

where $x=[x,y,z,\phi ,\theta ,\psi {]}^{T}$ is the state vector; $u=[F_{p}^{T},M_{p}^{T}{]}^{T}$ is the input vector; $G=ge_{3}$ represents the gravitational term; C denotes the know nonlinear terms caused by gravity and Coriolis effects; B is the control effectiveness matrix; D denotes the lumped disturbance vector, and satisfies $|D_{i}|<d_{i}$, where each *d*_*i*_>0 is a known upper bound of the corresponding disturbance component *D*_*i*_.

**Remark 2.** The proposed WRBF-FONTSMC strategy effectively relaxes the standard yet conservative assumption in conventional sliding mode control (SMC) that the upper bounds *d*_*i*_ of the lumped disturbance D (where $|D_{i}|<d_{i}$) must be known a priori. This is achieved by employing a WRBF neural network to provide a real-time estimate $\hat{D}$ of the total disturbance, thereby eliminating the controller’s explicit dependence on the precise knowledge of *d*_*i*_.

By setting the desired state $x_{d}=[x_{d},y_{d},z_{d},\phi_{d},\theta_{d},\psi_{d}{]}^{T}$, the tracking error and its derivative are defined as:

$$e=x_{d}-x,\;\dot{e}={\dot{x}}_{d}-\dot{x}$$ (17)

The primary control objective of this work is to design a robust 6-DOF trajectory tracking controller to stabilize the vehicle subjected to the model uncertainties and external disturbances. The tracking errors given by Equation (17) are required to converge to zero in finite-time:

$$\lim_{t\to t_{f}}\left\|e\right\|=0,\;\lim_{t\to t_{f}}\left\|\dot{e}\right\|=0$$ (18)

## Controller design

**Fig 3:**
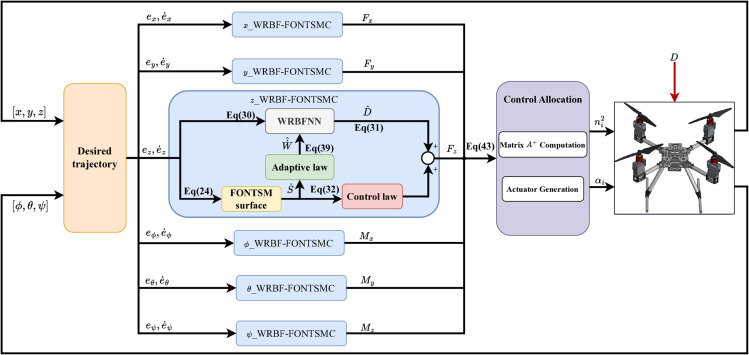
Control structure of system.

In this section, a fractional-order non-singular terminal sliding mode controller with WRBF disturbance estimation is designed for the proposed model (7), and its stability is rigorously analyzed. The primary control objective is to achieve accurate a 6-DOF reference trajectory tracking, encompassing both position and attitude. The controller leverages the system’s thrust vectoring capability to decouple, enabling implementation of independent control laws for each. These control laws calculate the necessary forces and torques to form the control wrench $u={\left[\begin{array}{cc} F_{p} & M_{p} \end{array}\right]}^{T}$ based on the position and attitude errors. To computer the actuator commands *n*_*i*_ and $\alpha_{i}$, the Moore-Penrose pseudo-inverse of the static allocation matrix is employed, as described in equation (12). The overall control structure is depicted in Fig 3, where the interconnections between the main controller components and their corresponding mathematical formulations (Eqs 24, 30, 31, 32, 40, 47) are highlighted for clarity.

### Introduction to fractional calculus

Fractional calculus generalizes integer-order calculus to cases where the order of differentiation or integration is a fraction. The commonly used basic operator in fractional calculus is denoted as ${\;}_{t_{0}}{𝒟}_{t}^{\alpha }$, and its intuitive expression is:

$${\;}_{t_{0}}{𝒟}_{t}^{\alpha }=\left\{ \begin{array}{cc} \frac{d^{\alpha }}{dt^{\alpha }}, & \text{Re}(\alpha )>0\\ 1, & \text{Re}(\alpha )=0\\ \int_{t_{0}}^{t}(d\tau {)}^{-\alpha }, & \text{Re}(\alpha )<0 \end{array}\right.$$ (19)

In the above expression, α represents the order; Re(α) denotes its real part; *t*_0_ and *t* are the lower and upper limits of the integral operator. In this article, $D^{\alpha }$ is used instead of ${\;}_{t_{0}}{𝒟}_{t}^{\alpha }$.

Currently, three definitions of fractional calculus are widely used in control theory: Grunwald-Letnikov(GL), Riemann-Liouville (RL), and Caputo [42]. This article adopts the Grunwald-Letnikov(GL) definition, which is defined as follows.

**Definition 1.** The Grunwald-Letnikov fractional calculus is defined as:

 t0Dtαf(t)=limh→0h−α∑j=0[t−t0h](−1)j(αj)f(t−jh) (20)

where [*x*] represents the integer part of *x*, *h* is the calculation step size, and (αj) is the binomial coefficient. The expression for the binomial coefficient is as follows:

(αj)=Γ(α+1)Γ(j+1)Γ(α−j+1) (21)

**Property 1** [53]. The fractional-order integration or differentiation calculus is a linear operation:

$$D^{\alpha }(ax_{1}(t)+bx_{2}(t))=aD^{\alpha }x_{1}(t)+bD^{\alpha }x_{2}(t)$$ (22)

**Property 2** [53]. For n∈ℕ and $\alpha \in {\mathbb{R}}^{+}$, the following equality holds for the fractional derivative operator $D^{\alpha }x(t)$ with $\frac{d^{n}}{dt^{n}}$

$$\frac{d^{n}}{dt^{n}}\left(D^{\alpha }x(t)\right)=D^{\alpha +n}x(t)$$ (23)

**Remark 3.** This work utilizes the Grünwald-Letnikov (GL) definition of the fractional derivative primarily for its computational advantages in digital control. The GL formulation is inherently discrete, expressed as a convolution that can be efficiently computed online using a recursive algorithm. This approach leverages a finite history of past values, significantly reducing the computational burden at each time step. In contrast, the Caputo definition often requires the more intensive numerical evaluation of an integral. For the real-time control of our tilt-rotor UAV, where the fractional-order derivative $D^{\alpha }e_{i}(t)$ in the sliding surface (Eq 24) must be updated rapidly, the GL definition provides a more feasible and efficient implementation without sacrificing the performance benefits of fractional-order control.

### FONTSM controller design

To ensure accurate and fast tracking under the presence of external disturbances and model uncertainties, a FONTSMC scheme is developed for all six degrees of freedom of the tiltable quadrotor system. According to [54], each channel i∈{1,2,...,6}, the sliding surface is defined as:

$$s_{i}={𝒟}^{\alpha }e_{i}+\frac{1}{\beta_{i}}{\dot{e}}_{i}^{\frac{p}{q}}$$ (24)

where α∈(0,1) is the fractional order, $\beta_{i}>0$ is a designed constant, and $\frac{p}{q}\in (1,2)$ with *p*, *q* being odd positive integers, ${\dot{e_{i}}}^{\frac{p}{q}}=sign({\dot{e}}_{i}){\left|{\dot{e}}_{i}\right|}^{\frac{p}{q}}$.

By differentiating Equation (24) with respect to time and utilizing Equations (7) and (17), the following expression is obtained:

$${\dot{s}}_{i}=D^{\alpha +1}e_{i}+\frac{p}{\beta_{i}q}{\dot{\left|e_{i}\right|}}^{\frac{p}{q}-1}\left({\ddot{x}}_{d,i}-C_{i}-(Bu{)}_{i}-D_{i}\right)$$ (25)

The exponential reaching law is introduced and designed as follows to prevent singularity issue:

$${\dot{s}}_{i}=-\frac{p}{\beta_{i}q}{\dot{\left|e_{i}\right|}}^{\frac{p}{q}-1}\left(\eta_{i}\;sign(s_{i})+k_{i}s_{i}\right)$$ (26)

where $\eta_{i},k_{i}>0$ are design parameters that control the convergence speed and damping of the system.

**Remark 4.** The exponential reaching law is selected considering the integrated control architecture. The primary role of disturbance rejection is delegated to the WRBF neural network, which accurately estimates and compensates for lumped uncertainties in real-time. Consequently, the reaching law is primarily responsible for governing the convergence dynamics, a task for which the exponential law is well-suited due to its simplicity and effectiveness. This approach, combined with the continuous sigmoid function and the chattering-suppression properties of fractional-order calculus, forms a comprehensive strategy that ensures smooth and robust performance without the added complexity of higher-order algorithms like super-twisting.

By combining Equation (25) with the fractional-order exponential reaching law Equation (26), the control law is derived as follows:


$$u_{i}=\frac{1}{B_{i}}({\ddot{x}}_{d,i}+\eta_{i}\;sign(s_{i})+k_{i}s_{i}+\frac{\beta_{i}q}{p}{𝒟}^{\alpha }{\dot{\left|e_{i}\right|}}^{2-\frac{p}{q}}sign({\dot{e}}_{i})$$

$$\;\;-C_{i}-{\hat{D}}_{i})$$ (27)

where ${\hat{D}}_{i}$ is the estimated disturbance provided by the WRBF neural network (to be detailed in Section Wavelet RBF Neural Network.

Finally, the full control input vector is composed as:

$$u={\left[\begin{array}{cccc} u_{1} & u_{2} & \ldots & u_{6} \end{array}\right]}^{T}$$ (28)

**Remark 2.** The discontinuity of the sign(·) function often causes significant chattering issues [55]. To resolve this, we propose substituting the sign(x) function with a continuous sigmoid(x) function. This substitution effectively reduces chattering [56]. The definition of the sigmoid(x) function is provided below:

$$sigmoid(x)=\frac{2}{1+e^{-bx}}-1$$ (29)

Here, *b* is an adjustable parameter that controls the steepness of the sigmoid function, allowing for fine-tuning of the control input’s smoothness.

### Wavelet RBF neural network

To estimate the unknown lumped disturbance $D\in {\mathbb{R}}^{6}$ in real time, an adaptive Wavelet Radial Basis Function Neural Network (WRBFNN) is introduced as the core disturbance observer. The network output $\hat{D}$ serves as an estimate of D, which is a directly injected into the control law for active cancellation

**Fig 4:**
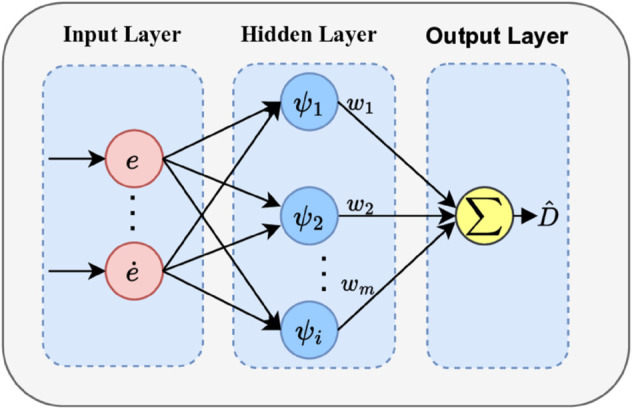
Structure of WRBF neural network.

The WRBFNN is a three-layer feedforward network composed of an input layer, hidden layer, and output layer, as shown in Fig 4.

**Input Layer:** The input vector is $x=[e^{T},{\dot{e}}^{T}{]}^{T}\in {\mathbb{R}}^{12}$, $e\in {\mathbb{R}}^{6}$ representing the the position and attitude tracking errors and their derivatives. This choice provides the network with direct access to the system’s dynamic performance deviation, enabling it to generate a state-dependent disturbance estimate.

**Hidden Layer:** Each neuron utilizes a Mexican Hat wavelet function as its activation:

$$\psi_{i}(x)=\left(1-{\left(\frac{\left\|x-c_{i}\right\|}{b_{i}}\right)}^{2}\right)\exp\left(-\frac{1}{2}{\left(\frac{\left\|x-c_{i}\right\|}{b_{i}}\right)}^{2}\right)$$ (30)

where $c_{i}$ is the center and *b*_*i*_ is the scale parameter for the *i*-th neuron. Compared to the conventional Gaussian function, the Mexican Hat wavelet offers superior local feature extraction capabilities and faster convergence, leading to enhanced approximation accuracy and noise robustness. [57]

**Output Layer:** The output of the network is the estimated lumped disturbance vector, calculated as a linear combination of the activated wavelet functions:

$$\hat{D}={\hat{W}}^{T}\Psi (x)={\left[\begin{array}{c} {\hat{f}}_{1},{\hat{f}}_{2},{\hat{f}}_{3},{\hat{m}}_{1},{\hat{m}}_{2},{\hat{m}}_{3} \end{array}\right]}^{T}$$ (31)

This WRBFNN structure leverages the Mexican Hat wavelets as its activation function, building upon the framework established in [57]. In our work, this network is specifically tailored and integrated as an adaptive disturbance observer within the control loop. Here, $\hat{W}\in {\mathbb{R}}^{N\times 6}$ is the estimated weight matrix; $\Psi (x)\in {\mathbb{R}}^{N}$ is the vector of Mexican Hat basis functions; ${\hat{f}}_{i}$ and ${\hat{m}}_{i}$ represent the estimated force and moment components, respectively.

By integrating the estimated disturbance from Equation (31) into the FONTSMC control law, we derive the complete control input $u\in {\mathbb{R}}^{6}$ as:

$$u=\frac{1}{B}({\ddot{x}}_{d}+\eta \;sigmoid(s)+ks+\frac{\beta q}{p}{𝒟}^{\alpha }{\dot{\left|e\right|}}^{2-\frac{p}{q}}sign(\dot{e})-C-\hat{D})$$ (32)

This six-dimensional control input $u=[F_{p}^{T},M_{P}^{T}{]}^{T}$ is composed of desired force vector $F_{p}$ and moment $M_{p}$. They are respectively expressed as:

$$F_{p}=mR_{g}^{b}({\ddot{P}}_{d}+\eta \;sigmoid(s)+ks+\frac{\beta q}{p}{𝒟}^{\alpha }{\dot{P}}_{e}^{2-\frac{p}{q}}sign({\dot{P}}_{e})-ge_{3}-\hat{f})$$ (33)

$$M_{p}=J_{b}({\ddot{𝛩}}_{d}+\eta \;sigmoid(s)+ks+\frac{\beta q}{p}{𝒟}^{\alpha }{\dot{𝛩}}_{e}^{2-\frac{p}{q}}sign({\dot{𝛩}}_{e})+\omega \times J_{b}\omega -\hat{m})$$ (34)

where $\hat{f}=[{\hat{f}}_{1},{\hat{f}}_{2},{\hat{f}}_{3}{]}^{T}$, $\hat{m}=[{\hat{m}}_{1},{\hat{m}}_{2},{\hat{m}}_{3}]$ are estimated force and moments vector; ${\ddot{P}}_{d},{\ddot{𝛩}}_{d}$ are the desired accelerations for position and attitude.

### Stability analysis

**Lemma 1.** [58] Suppose there exists a positive definite Lyapunov function *V*(*x*) that satisfies the inequality $\dot{V}(x)+k_{1}V(x)+k_{2}V^{k_{3}}(x)\leq 0,$ where $k_{1},k_{2}>0$ and 0<*k*_3_<1. Let $V_{0}(x)$ denote the initial value of *V*(*x*). Then the system state converges to zero in finite time, and the upper bound of the convergence time *T* is given by:

$$T\leq \frac{1}{k_{1}(1-k_{3})}\ln\;\left(\frac{k_{1}V_{0}^{1-k_{3}}(x)+k_{2}}{k_{2}}\right)$$ (35)

**Theorem 1.** For the nonlinear system described by Equation (7), under the proposed control law (Equation (32)) which integrates the FONTSMC with WRBFNN using the Mexican Hat basis function, all system states converge to zero in finite time.

**Proof of Theorem 1.** Define the Lyapunov function for each channel i∈{1,2,…,6} as:

$$L=\frac{1}{2}s_{i}^{2}+\frac{1}{2}\gamma_{i}{\tilde{W}}_{i}^{T}{\tilde{W}}_{i},\;\gamma_{i}>0$$ (36)

where *s*_*i*_ is the sliding surface, ${\tilde{W}}_{i}=W_{i}-{\hat{W}}_{i}$ is the weight estimation error, and $\gamma_{i}>0$ is a positive constant.

Taking its derivative yields:

$$\dot{L}=s_{i}{\dot{s}}_{i}+\gamma_{i}{\tilde{W}}_{i}^{T}{\dot{\tilde{W}}}_{i}$$ (37)

Substituting the control law (Equation 32) into the dynamic expression for ${\dot{s}}_{i}$, we get:

$${\dot{s}}_{i}=-\frac{p}{\beta_{i}q}{\left|{\dot{e}}_{i}\right|}^{\frac{p}{q}-1}\left({\tilde{D}}_{i}+\eta_{i}\;sigmoid(s_{i})+k_{i}s_{i}\right)$$ (38)

where ${\tilde{D}}_{i}=D_{i}-{\hat{D}}_{i}={\tilde{W}}_{i}^{T}\Psi (x)+\varepsilon_{i}$ denotes the approximation error of the WRBFNN, and $\varepsilon_{i}$ is bounded.

Substituting (38) into (37) gives:

$$\dot{L}=-\frac{p}{\beta_{i}q}{\left|{\dot{e}}_{i}\right|}^{\frac{p}{q}-1}s_{i}\left({\tilde{W}}_{i}^{T}\Psi (x)+\varepsilon_{i}+\eta_{i}sigmoid(s_{i})+k_{i}s_{i}\right)+\gamma_{i}{\tilde{W}}_{i}^{T}{\dot{\tilde{W}}}_{i}$$ (39)

Choose the adaptive update law as:

$${\dot{\hat{W}}}_{i}=\frac{1}{\gamma_{i}}\frac{p}{\beta_{i}q}{\left|{\dot{e}}_{i}\right|}^{\frac{p}{q}-1}\Psi (x)s_{i}$$ (40)

Then, the Lyapunov derivative becomes:

$$\dot{L}=-\frac{p}{\beta_{i}q}{\left|{\dot{e}}_{i}\right|}^{\frac{p}{q}-1}\left(\varepsilon_{i}s_{i}+\eta_{i}|s_{i}|+k_{i}s_{i}^{2}\right)$$ (41)

Assuming $1<\frac{p}{q}<2$, $\beta_{i}>0$, and $\varepsilon_{i}$ bounded, it follows that $\dot{L}\leq 0$, thus satisfying the Lyapunov stability condition. Therefore, the closed-loop system is globally asymptotically stable.

To establish finite-time convergence, consider the dynamics of the sliding variable *s*_*i*_. From (38) and the boundedness of ${\tilde{D}}_{i}$, there exists a positive constant $\rho_{i}$ such that $|{\tilde{D}}_{i}|\leq \rho_{i}$. Since $sig(s_{i})$ is a smooth approximation of $sign(s_{i})$ satisfying $|sig(s_{i})-sign(s_{i})|\leq \delta_{s}$ (where $\delta_{s}$ is small), and noting that $|{\dot{e}}_{i}{|}^{p/q-1}>0$, the reaching dynamics can be upper bounded by:

$${\dot{s}}_{i}\leq -\frac{p}{\beta_{i}q}|{\dot{e}}_{i}{|}^{\frac{p}{q}-1}\left[(\eta_{i}-\rho_{i})sign(s_{i})+k_{i}s_{i}\right]$$ (42)

Defining the positive constants: $\kappa_{1}=\frac{p}{\beta_{i}q}|{\dot{e}}_{i}{|}^{\frac{p}{q}-1}(\eta_{i}-\rho_{i}),\;\kappa_{2}=\frac{p}{\beta_{i}q}|{\dot{e}}_{i}{|}^{\frac{p}{q}-1}k_{i}$, we obtain the differential inequality:

$${\dot{s}}_{i}\leq -\kappa_{1}\;sign(s_{i})-\kappa_{2}|s_{i}|$$ (43)

Equation (43) corresponds to a standard finite-time stable system [58]. By integrating this inequality, the finite-time settling bound of *s*_*i*_ can be derived as:

$$T_{s}\leq \frac{1}{\kappa_{2}}\ln\;\left(1+\frac{\kappa_{2}}{\kappa_{1}}|s_{i}(0)|\right)$$ (44)

Hence, the sliding variable *s*_*i*_ converges to zero within the finite time *T*_*s*_. Once the system reaches the manifold *s*_*i*_ = 0, the sliding surface dynamics reduce to:

$${𝒟}^{\alpha }e_{i}+\frac{1}{\beta_{i}}{\dot{e}}_{i}^{p/q}=0$$ (45)

which satisfies the finite-time convergence condition according to Lemma 1. Therefore, both *e*_*i*_ and ${\dot{e}}_{i}$ converge to zero in finite time. ∎

**Remark 5.** The above proof demonstrates that the proposed WRBF-FONTSMC scheme guarantees finite-time stability. The upper bound of the settling time *T*_*s*_ for *s*_*i*_ to reach zero from its initial value *s*_*i*_(0) is explicitly given by:

$$T_{s}\leq \frac{\beta_{i}q}{pk_{i}|{\dot{e}}_{i}{|}^{\frac{p}{q}-1}}\ln\;\left(1+\frac{k_{i}}{\eta_{i}-\rho_{i}}|s_{i}(0)|\right)$$ (46)

where $\rho_{i}$ denotes the upper bound of the neural network approximation error ${\tilde{D}}_{i}$. This expression provides a conservative estimate of the convergence speed, which can be tuned through the controller parameters *k*_*i*_, $\eta_{i}$, and $\beta_{i}$. During the reaching phase, $|{\dot{e}}_{i}{|}^{p/q-1}$ is slowly varying and can be approximated as constant, ensuring the analytical validity of (46).

### Control allocation

In the context of control allocation, the six-degree-of-freedom (6-DOF) control wrench u is mapped to eight actuator commands. This problem is typically challenging due to the non-uniqueness of solutions arising from the system’s over-actuation. Furthermore, without proper selection, the allocation matrix can nonlinearly depend on the controllable inputs, necessitating a computationally expensive optimization process. To address these challenges, we propose a linear solution that first transforms the forces and moments generated by each rotor into vertical and lateral components, as shown in (12). Since the allocation matrix A is static, the vertical and lateral components 𝛺 can be efficiently computed by inverting (12) as follows:

$$𝛺=A^{†}[\begin{array}{c} F_{p}\\ M_{p} \end{array}]$$ (47)

where $A^{†}$ is the Moore–Penrose pseudo-inverse matrix of A.

$$A^{†}=[\begin{array}{cccccc} 0 & \frac{1}{2} & 0 & 0 & 0 & \frac{L}{4(L^{2}+c^{2})}\\ 0 & -\frac{c}{2L} & \frac{1}{4} & 0 & -\frac{1}{2L} & \frac{c}{4(L^{2}+c^{2})}\\ -\frac{1}{2} & 0 & 0 & 0 & 0 & \frac{L}{4(L^{2}+c^{2})}\\ 0 & 0 & \frac{1}{4} & \frac{1}{2L} & 0 & -\frac{c}{4(L^{2}+c^{2})}\\ 0 & -\frac{1}{2} & 0 & 0 & 0 & \frac{L}{4(L^{2}+c^{2})}\\ 0 & \frac{c}{2L} & \frac{1}{4} & 0 & \frac{1}{2L} & \frac{c}{4(L^{2}+c^{2})}\\ \frac{1}{2} & 0 & 0 & 0 & 0 & \frac{L}{4(L^{2}+c^{2})}\\ 0 & 0 & \frac{1}{4} & -\frac{1}{2L} & 0 & -\frac{c}{4(L^{2}+c^{2})} \end{array}]$$ (48)

And then the tilting angles and motor speeds can be computed directly.


$$n_{i}^{2}=\frac{1}{k_{f}}\sqrt{\Omega_{j-1}^{2}+\Omega_{j}^{2}},\;(j=2i)$$

$$\alpha_{i}=atan2(\Omega_{j-1},-\Omega_{j}),\;(j=2i)$$ (49)

This approach allows for the utilization of a static allocation matrix that remains independent of the controllable inputs, thereby enabling a computationally efficient solution. By eliminating the need for nonlinear optimization, the method significantly reduces the computational complexity typically associated with control allocation problems.

To ensure that the control allocation results are physically realizable, actuator constraints were explicitly modeled in the simulation. The tilting servo angles of each rotor were restricted to $\left[20^{\circ },160^{\circ }\right]$, consistent with the mechanical range of the tilt-rotor joints. The generated thrust commands were bounded as $8\;N\leq F_{i,z}\leq 40\;N$ for vertical lift, and the lateral thrust components were saturated within ±6 N to reflect aerodynamic limitations. Similarly, the control moments were constrained by $M_{d}=\text{sat}(M_{d},\pm 1.5\;N\cdot m)$ to guarantee feasible torque generation.

These limits were implemented via nonlinear saturation functions applied after the Moore–Penrose pseudo-inverse allocation. Whenever the commanded values exceeded their physical boundaries, they were projected back into the admissible range.

### Parameter tuning

Given that the control performance in practical applications is susceptible to factors like model uncertainties and actuator limitations, the parameter set must be carefully engineered to ensure reliable operation. In this section, we elucidate the function of each parameter and offer a set of well-tuned, suggested values that have been verified to yield robust performance across various flight scenarios.

1) Selections of *p*, *q*, β: These parameters define the fractional-order non-singular terminal sliding surface in Eq (24). The ratio p/q∈(1,2) ensures finite-time convergence without singularity, with larger values accelerating convergence but potentially increasing control effort. Parameter β weights the terminal attractor term, where increasing β enhances dynamic response but may amplify noise. Recommended values are *p* = 9, *q* = 7, and β=1/5 per channel requirements.

2) Selections of *k*, η: These parameters govern convergence and disturbance rejection. Gain *k* controls sliding variable convergence, where larger values accelerate response but may cause oscillations. Switching gain η provides robustness but causes chattering if excessive. The sign function is replaced by a continuous sigmoid function Eq (29) to mitigate this effect. Recommended ranges are *k* = 10 and η=5.

3) Selections of α: The fractional order α in Eq (24) introduces memory effects that smooth control signals and reduce chattering. Higher values improve smoothness but may slow initial response. Empirical results suggest α=0.5 provides an effective balance between response speed and signal smoothness.

4) Selections of *c*_*ij*_, *b*_*j*_, γ: The WRBF neural network employs Mexican Hat wavelets Eq (30) for disturbance estimation in the control law Eq (27). The centers *c*_*ij*_ are uniformly distributed over [–1, 1] to cover the normalized error range. Widths *b*_*j*_ = 3 balance localization and generalization, while the learning rate γ=5 ensures stable weight adaptation in Eq (39) across all channels.

### Theoretical comparison and analysis

Having established the finite-time stability and boundedness of the proposed WRBF-FONTSMC controller, this section further compares its theoretical characteristics with other existing control schemes. A detailed comparison of key control attributes is summarized in Table 1.

**Table 1:** Theoretical comparison among different control schemes.

Controllers	Characteristics	Advantages	Disadvantages
NN-PID	Adaptive tuning Feedback-based Gradient optimization	Easy implementation Wide applications Self-tuning	Asymptotic stability Slow response No robustness guarantee
NTSMC	Robust control Switching-based Finite-time stability	Simple structure Fast convergence Strong robustness	Severe chattering Needs disturbance bound Conservative gains
FONTSMC	Fractional-order Smooth switching Finite-time stability	Reduced chattering Memory effects Extra tuning parameters	Complex implementation Conservative design Computationally heavy
RBF-FONTSMC	Adaptive-robust Neural compensation Feedforward control	Reduced gains Better accuracy Online learning	Approximation errors Training required Parameter sensitivity
WRBF-FONTSMC (proposed)	Wavelet-enhanced Precise compensation Noise-resistant	Superior accuracy Fast learning Strong robustness	High computation Complex tuning Memory intensive

Table 1 provides a theoretical comparison of five controllers, including NN-PID, NTSMC, FONTSMC, RBF-FONTSMC, and the proposed WRBF-FONTSMC. The NN-PID offers simple implementation but limited robustness, while NTSMC achieves finite-time convergence at the cost of chattering due to its discontinuous control law. The FONTSMC introduces fractional-order dynamics to improve convergence speed and memory characteristics. The RBF-FONTSMC enhances disturbance compensation by using a Gaussian RBF neural estimator, though its global approximation can lead to slower adaptation in nonstationary conditions. In contrast, the proposed WRBF-FONTSMC integrates a wavelet-based neural estimator with a fractional-order nonsingular terminal sliding surface and a smooth sigmoid reaching law. This synergistic design achieves fast finite-time convergence, improved robustness, and chattering-free performance, making it more suitable for six-degree-of-freedom UAV control under complex disturbances.

## Simulation and results analysis

**Fig 5:**
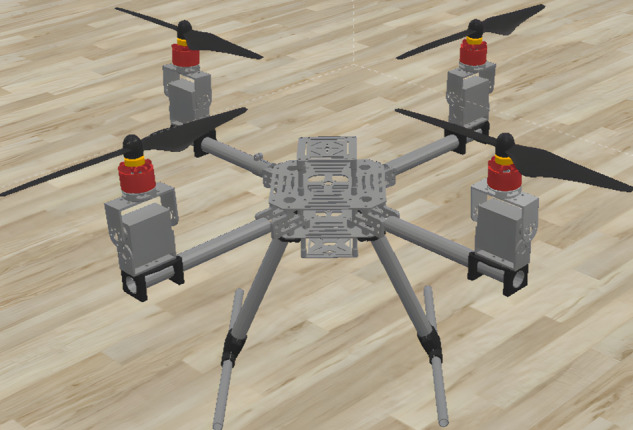
The tiltable quadrotor model.

This section presents numerical simulations to validate the effectiveness and superiority of the proposed WRBF-FONTSMC control strategy. Simulations were performed in CoppeliaSim using MATLAB/Simulink for control implementation, as illustrated in Fig 5. Three experimental scenarios were designed: fixed-attitude trajectory tracking, fixed-point rotation, and wind disturbance tests, to systematically evaluate the controller’s tracking accuracy, decoupling capability, and robustness.

Four representative benchmark controllers were selected for comprehensive performance evaluation: NTSMC assesses the contribution of fractional-order operators, FONTSMC verifies the necessity of neural network compensation, RBF-FONTSMC compares the advantages of wavelet functions, and NN-PID serves as a reference from different control frameworks. The experimental design comprises three test scenarios: fixed-attitude trajectory tracking examines basic tracking performance, fixed-point rotation validates full-actuation control capability, and wind disturbance tests evaluate anti-interference performance. All controllers utilized identical model parameters and test conditions, with parameters optimized through systematic tuning to ensure fair comparison.

The experiments used a tilt-rotor quadrotor platform with physically measured parameters. Total mass and arm length were obtained through direct measurement, inertia tensor was calculated based on SolidWorks mass property analysis, and aerodynamic coefficients were derived from propeller technical data sheets, with specific parameters listed in Table 2. Controller parameters were systematically optimized as detailed in Table 3.

**Table 2:** Quadcopter physical and aerodynamic parameters. This table lists the core mechanical and aerodynamic properties used in the simulation.

Parameter	Value (Unit)	Source/Method
*g*	9.810 (m/s^2^)	Standard
*d*	0.240 (m)	Direct measurement
*C*_*T*_	$1.4865\times 10^{-7}$ (N/RPM^2^)	Manufacturer datasheet
*C*_*M*_	$2.9250\times 10^{-9}$ (N/RPM^2^)	Derived from *C*_*T*_
*J*_*xx*_	0.365 (kg·m^2^)	SolidWorks mass analysis
*J*_*yy*_	0.365 (kg·m^2^)	SolidWorks mass analysis
*J*_*zz*_	0.774 (kg·m^2^)	SolidWorks mass analysis
*m*	1.605 (kg)	Direct measurement

Table notes: *C*_*T*_ and *C*_*M*_ represent the thrust and moment coefficients; $J_{xx},J_{yy},J_{zz}$ are the principal moments of inertia; *d* is the arm length, and *m* is the total mass.

**Table 3:** Control parameters in the simulation.

Parameter	NN-PID	NTSM	FONTSM	RBF-FONTSM	WRBF-FONTSM
p(x,y,z,ϕ,θ,ψ)	–	9,9,9 9,9,9	7,7,7 7,7,7	9,9,9,9,9,9	9,9,9,9,9,9
q(x,y,z,ϕ,θ,ψ)	–	7,7,7 7,7,7	7,7,7 7,7,7	5,5,5,5,5,5	7,7,7,7,7,7
β(x,y,z,ϕ,θ,ψ)	–	1/3,1/3,1/5 1/2,1/2,1/2	1/5,1/5,1 1/5,1/5,1/5	1/5,1/5,1/5,1/2,1/2,1/2	1/5,1/5,1/5,1/3,1/3,1/3
k(x,y,z,ϕ,θ,ψ)	–	10,10,10 3,3,3	10,10,20 10,10,10	20,20,20,10,6,6	10,10,10,10,10,10
η(x,y,z,ϕ,θ,ψ)	–	15,15,15 6,6,6	10,10,20 10,10,10	6,6,6,10,6,6	6,6,6,6,6,6
α(x,y,z,ϕ,θ,ψ)	–	–	0.6,0.6,0.6 0.5,0.5,0.5	0.6,0.6,0.6,0.5,0.5,0.5	0.6,0.6,0.6,0.5,0.5,0.5
γ(x,y,z,ϕ,θ,ψ)	5,5,5,5,5,5	–	–	5,5,30,10,10,10	5,15,15,15,15,15
$b_{j}(x,y,z,\phi ,\theta ,\psi )$	3,3,3,3,3,3	–	–	3,3,3,3,3,3	3,3,3,3,3,3
$c_{ij}(x,y,z,\phi ,\theta ,\psi )$	$\left(\begin{array}{ccccc} -1 & -0.5 & 0 & 0.5 & 1\\ -1 & -0.5 & 0 & 0.5 & 1 \end{array}\right)$	–	–	$\left(\begin{array}{ccccc} -1 & -0.5 & 0 & 0.5 & 1\\ -1 & -0.5 & 0 & 0.5 & 1 \end{array}\right)$	$\left(\begin{array}{ccccc} -1 & -0.5 & 0 & 0.5 & 1\\ -1 & -0.5 & 0 & 0.5 & 1 \end{array}\right)$
Kp(x,y,z,ϕ,θ,ψ)	15,20,15,15,15,10	–	–	–	–
Ki(x,y,z,ϕ,θ,ψ)	3,5,3,3,5,3	–	–	–	–
Kd(x,y,z,ϕ,θ,ψ)	8,10,6,8,8,10,10	–	–	–	–

### Fixed-attitude trajectory tracking

As a fundamental test of tracking performance, this experiment evaluates the controller’s ability to follow a 3D trajectory while maintaining a fixed attitude. The desired trajectory is:


$$\left[X_{d},\;Y_{d},\;Z_{d}]=[0.5\;\sin(t),\;0.5\;\cos(t),\;1+0.5\;\sin(t)\right]$$

$$\left[\phi_{d},\;\theta_{d},\;\varphi_{d}\right]=[30^{\circ },\;30^{\circ },\;30^{\circ }]$$

**Fig 6:**
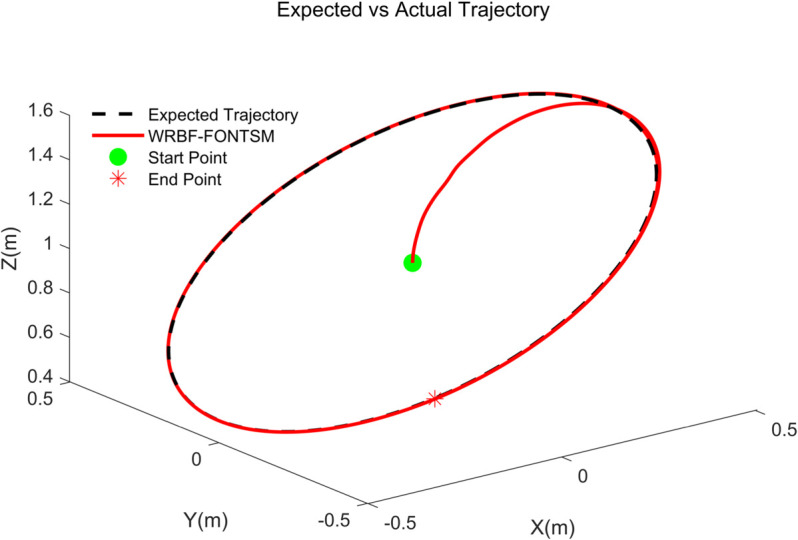
3D trajectory tracking.

**Fig 7:**
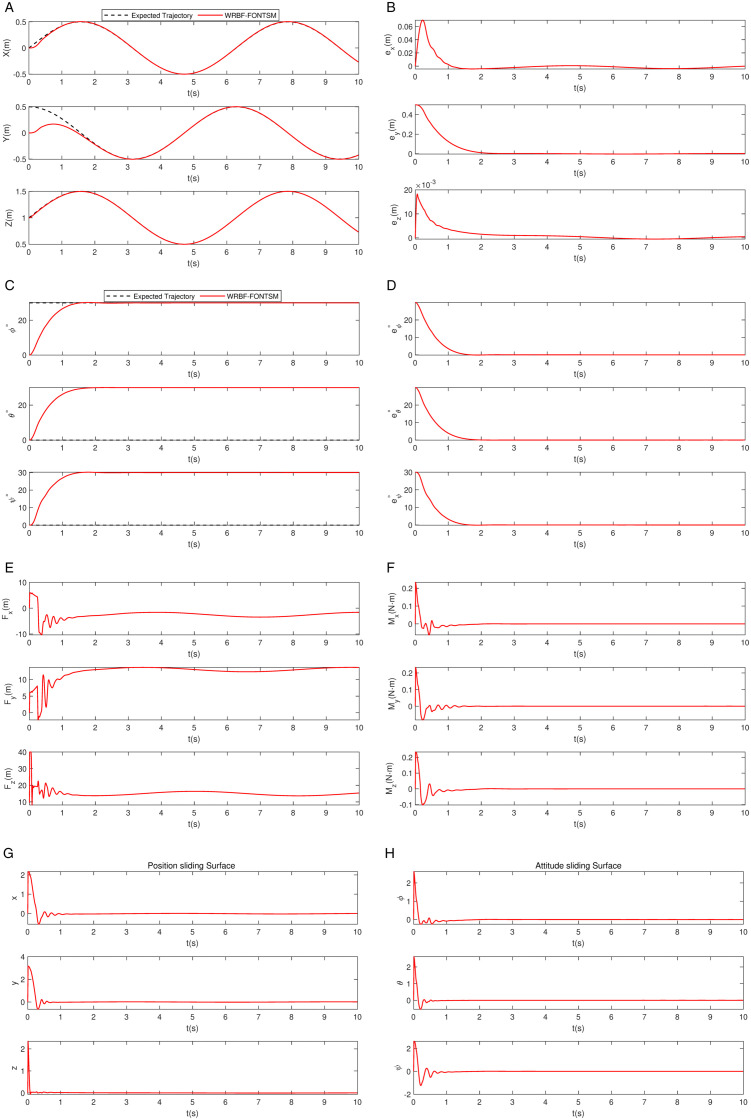
Trajectory tracking with fixed attitude. a: Position tracking. b: Position tracking error. c: Attitude tracking. d: Angular tracking errors. e: Control force. f: Control moment. g: Position sliding surface. h: Attitude sliding surface.

The proposed WRBF-FONTSMC controller demonstrates exceptional tracking performance in this basic test. As shown in Figs 6 and 7a, WRBF-FONTSMC accurately tracks the desired trajectory. The position error in Fig 7b converge rapidly within 2 seconds. Fig 7c shows perfect attitude maintenance at $\left[\phi_{d},\;\theta_{d},\;\varphi_{d}\right]=[30^{\circ },\;30^{\circ },\;30^{\circ }]$, with attitude errors remaining near zero in Fig 7d. The control forces and moments, presented in Fig 7e and 7f, display smooth profiles with no noticeable high-frequency chattering, highlighting the combined advantages of fractional-order operators and neural network compensation. Accordingly, the sliding surfaces in Figs 7g and 7h converge to zero within about 2 seconds and remain stable afterwards. These results collectively confirm that the WRBF-FONTSMC controller achieves fast convergence, minimal chattering, and strong robustness in both position and attitude control.

Based on the quantitative analysis in Table 4, the WRBF-FONTSMC controller demonstrates outstanding performance. In terms of convergence speed, all channels achieve convergence within 1.8 seconds, with the Z-axis responding particularly rapidly (0.0003 s). Regarding control quality, the overshoot in all channels remains below 1%, indicating a smooth and oscillation-free system response. Furthermore, the controller achieves remarkably high steady-state accuracy: the steady-state position error reaches as low as the order of 10^−4^ m, while the steady-state attitude error remains stable at the order of 10^−3^ rad. This series of data fully confirms that the control scheme ensures both rapid dynamic response and high control precision and stability.

**Table 4:** Key performance indexes of fix-attitude trajectory tracking.

Index	x	y	z	ϕ	θ	ψ
Convergence Time (s)	0.6000	1.7900	0.0003	1.4200	1.5600	1.4000
Overshoot (%)	1	0.6	0.1	0.3	0.3	0.3
Steady-state error	0.002142	0.001686	0.000240	0.005772	0.002575	0.005687

### Fixed point rotation

**Fig 8:**
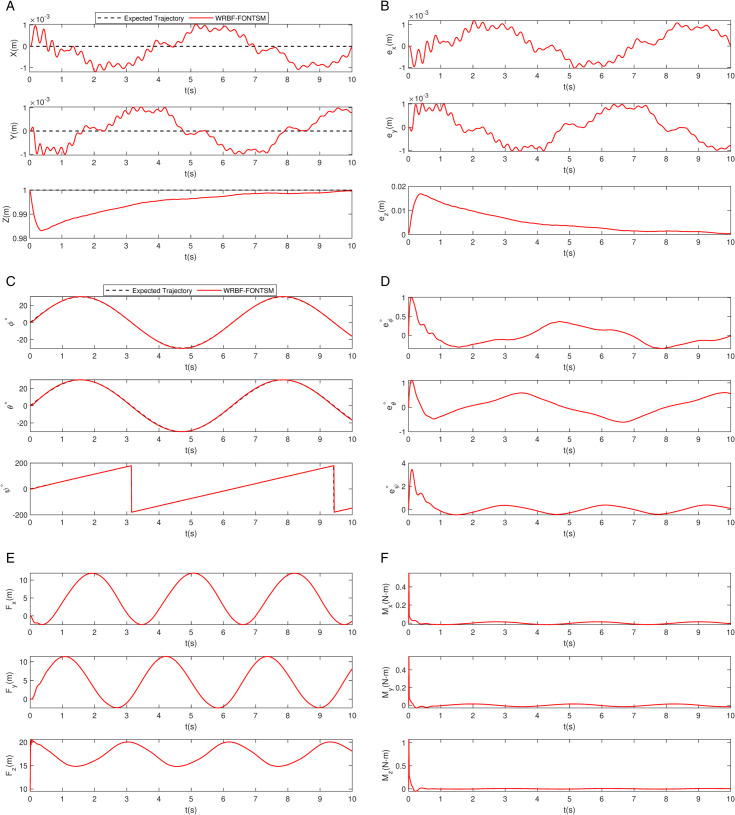
Fixed-point rotation performance. a: Position tracking. b: Position tracking error. c: Attitude tracking. d: Angular tracking errors. e: Control force. f: Control moment.

The tiltable quadcopter is capable of omnidirectional flight. To highlight its advantages over the traditional quadcopter design, a fixed-point rotation experiment is conducted. This experiment primarily examines the attitude tracking ability of the quadcopter during hovering. The simulation results are shown in Fig 8. The reference trajectory is:


$$\left[X_{d},\;Y_{d},\;Z_{d}]=[0,\;0,\;1\right]$$

$$\left[\phi_{d},\;\theta_{d},\;\varphi_{d}\right]=[\frac{\pi }{6}\sin(t),\;\frac{\pi }{6}\sin(t),\;t]$$

The proposed WRBF-FONTSMC controller demonstrates exceptional decoupling control capability in this challenging scenario. As shown in Fig 8a, WRBF-FONTSMC accurately maintains the designated position at [0,0,2] meters while performing simultaneous roll and pitch rotations following $\left[\phi_{d},\;\theta_{d},\;\varphi_{d}\right]=[\frac{\pi }{6}\sin(t),\;\frac{\pi }{6}\sin(t),\;t]$ radians. The position tracking errors in Fig 8b remain within strict bounds of ±0.02 m throughout the maneuver. Fig 8c shows precise attitude tracking of the reference commands, with attitude errors maintained within ±0.025 radians in Fig 8d. The control forces and moments in Fig 8e, 8f exhibit well-regulated characteristics without excessive actuation, demonstrating effective management of coupled dynamics through the integrated control framework.

To quantitatively assess the controller’s decoupling performance, key metrics summarized in Table 5. The controller exhibits notably fast convergence across all degrees of freedom, with convergence times not exceeding 1.5 seconds. In particular, the roll, the pitch, and yaw channels each stabilize within 0.3 seconds, indicating excellent dynamic response in attitude control. All overshoot values remain below 1.6%, reflecting well-damped and smooth transients. In terms of steady-state accuracy, the position errors in the x, y, and z axes are consistently on the order of 10^−4^ m, demonstrating high-precision tracking performance. Meanwhile, the steady-state attitude error remains stable on the order of 10^−1^ rad. In robotic dynamic control, this level of accuracy, combined with rapid convergence characteristics, is fully adequate to meet the core requirements for attitude stabilization in various complex tasks.

**Table 5:** Key performance indexes of fix point rotation.

Index	x	y	z	ϕ	θ	ψ
Convergence Time (s)	0.5	1.5	1.5	0.3	0.3	0.3
Overshoot (%)	0.1	0.1	1.6	1	1.6	0.2
Steady-state error	0.000771	0.000494	0.000610	0.190654	0.384125	0.262286

Subsequent simulation experiments further investigate the disturbance rejection and robustness of the WRBF-FONTSM controller.

### Anti-interference test

**Fig 9:**
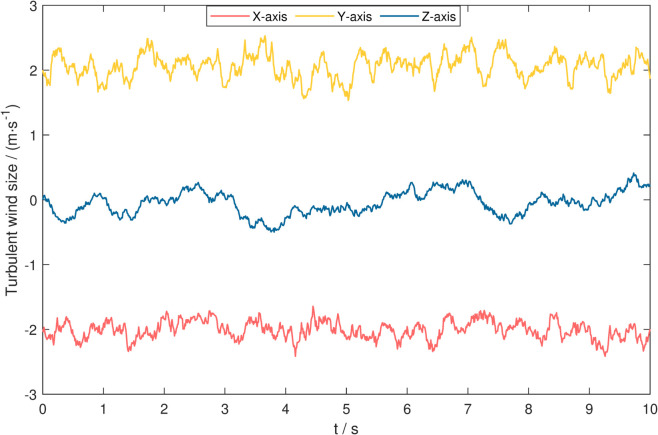
Turbulent wind field diagram.

This study establishes a composite wind field environment based on the Dryden turbulence model, generating disturbance signals that conform to atmospheric turbulence characteristics through shaped filtering of Gaussian white noise. It systematically evaluates the trajectory tracking accuracy, disturbance rejection capability, and dynamic response characteristics of five controllers under wind disturbance conditions. The experimental setup adopts a composite working condition combining a steady wind field of [2,1,0]m/s with random turbulence. Key turbulence parameters (including turbulence intensities *L*_*u*_, $L_{v}$, *L*_*w*_ and turbulence scales $\sigma_{u}$, $\sigma_{v}$, $\sigma_{w}$) are configured strictly according to the transfer function model in reference [59], with specific parameters provided in Table 6. The time-domain characteristics of the generated turbulent wind field are shown in Fig 9, providing a reliable disturbance environment benchmark for controller performance evaluation.


$$G_{u}(s)=\frac{K_{u}}{T_{u}s+1},\;K_{u}=\sigma_{u}\sqrt{\frac{L_{u}}{\pi v}},\;T_{u}=\frac{L_{u}}{v}$$

$$G_{v}(s)=\frac{K_{v}}{T_{v}s+1},\;K_{v}=\sigma_{v}\sqrt{\frac{L_{v}}{\pi v}},\;T_{v}=\frac{2L_{v}}{\sqrt{3}v}$$

$$G_{w}(s)=\frac{K_{w}}{T_{w}s+1},\;K_{w}=\sigma_{w}\sqrt{\frac{L_{w}}{\pi v}},\;T_{w}=\frac{2L_{w}}{\sqrt{3}v}$$ (50)

**Table 6:** Turbulent wind field parameters. Key parameter settings used for modeling the turbulent wind field.

Parameter	Value (Unit)
*L*_*u*_	75.639 (m)
$L_{v}$	37.820 (m)
*L*_*w*_	5.000 (m)
$\sigma_{u}$	0.982 (m/s)
$\sigma_{v}$	1.927 (m/s)
$\sigma_{w}$	0.500 (m/s)

The reference trajectory is:


$$\left[X_{d},\;Y_{d},\;Z_{d}]=[0.5\;\cos(2t),\;0.5\;\sin(2t),\;1+0.2t\right]$$

$$\left[\phi_{d},\;\theta_{d},\;\varphi_{d}\right]=[\frac{\pi }{6}\sin(t),\;\frac{\pi }{6}\sin(t),\;t]$$

**Fig 10:**
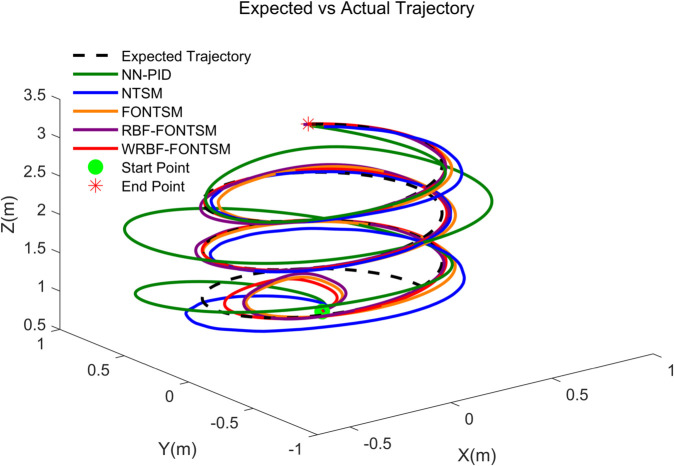
3D trajectory tracking.

**Fig 11:**
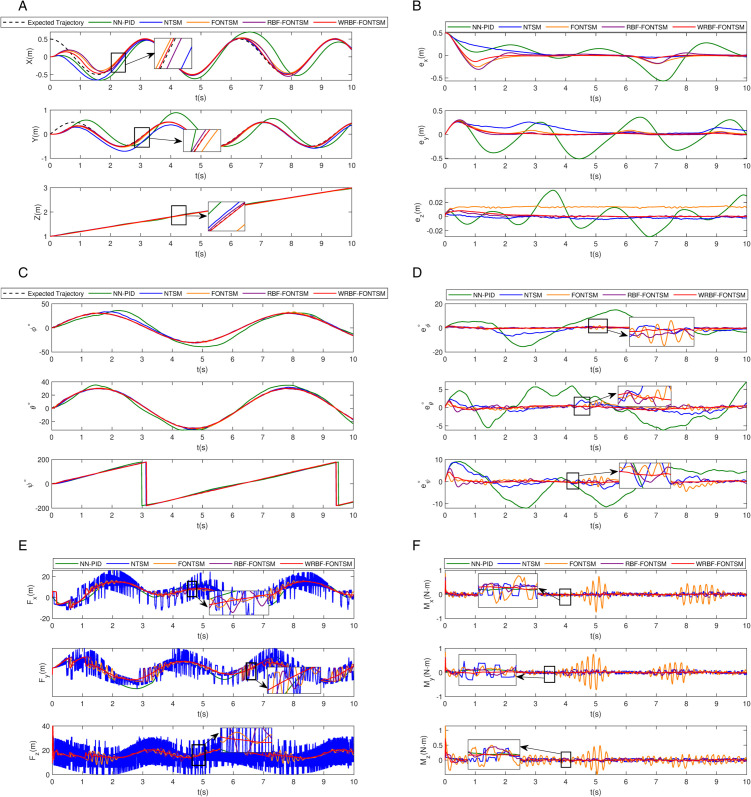
Trajectory tracking under the action of wind field. a: Position tracking. b: Position tracking error. c: Attitude tracking. d: Angular tracking errors. e: Control force. f: Control moment.

This simulation conducted a systematic performance evaluation of five typical controllers (NTSMC, FONTSMC, RBF-FONTSMC, WRBF-FONTSMC, and NN-PID) under combined steady-state wind field and turbulent conditions. The trajectory tracking results shown in Figs 10 and 11a demonstrate that: NN-PID exhibits significant steady-state error with incomplete convergence; NTSMC demonstrates initial deviation and slow convergence; both FONTSMC and RBF-FONTSMC achieve effective tracking but with overshoot, though RBF neural network integration significantly improves FONTSMC’s setting time. The proposed WRBF-FONTSMC achieves optimal comprehensive performance with the fastest settling time, minimal overshoot, and highest tracking accuracy.

Further analysis of position tracking errors in Fig 11b confirms these findings. WRBF-FONTSMC maintains position errors within ±0.01 meters with minimal oscillation, reducing error peaks by 50% compared to RBF-FONTSMC. Attitude control performance (Fig 11c) shows NN-PID produces smooth responses but with $10^{\circ }$ steady-state error; NTSMC confines errors within $5^{\circ }$ but with significant chattering; FONTSMC exhibits $\pm 2^{\circ }$ oscillations during gusts, which RBF compensation alleviates but doesn’t eliminate; while WRBF-FONTSMC achieves optimal tracking accuracy with smooth responses. The attitude error statistics in Fig 11d demonstrate WRBF-FONTSMC’s superior performance, strictly limiting errors to $\pm 0.5^{\circ }$.

The control inputs in Figs 11e and 11f reveal distinct performance characteristics. In terms of smoothness, both the NN-PID and the proposed WRBF-FONTSMC controllers generate signals free of chattering, while the NTSMC and other benchmark controllers exhibit significant or minor chattering, respectively. Regarding performance, the NN-PID’s capabilities are limited by its basic structure, making it far less effective than the WRBF-FONTSMC. Furthermore, the compensatory effect of the RBF and WRBF networks on the FONTSMC controller is clearly visible in Fig 11f around 5s and 8s. These observations collectively validate the superior control quality of the WRBF-FONTSMC approach.

Quantitative performance (Table 7) provide additional validation. WRBF-FONTSMC achieves the optimal RMSE of 0.2923, showing improvements of 91.2%, 70.0%, 32.3%, and 9.4% over NN-PID, NTSM, FONTSM, and RBF-FONTSM respectively. Its ITAE value of 3.7348 represents reductions of 97.4%, 78.2%, 51.7%, and 39.1% compared to the other controllers. While WRBF-FONTSMC’s ISU index 704 is slightly higher than FONTSM’s optimal value, it demonstrates reductions of 1.7% and 18.5% compared to NN-PID and NTSM respectively. Most notably, WRBF-FONTSMC maintains similar control energy consumption to RBF-FONTSM while achieving significantly improved control accuracy, highlighting the unique advantages of wavelet radial basis functions in control performance optimization.

**Table 7:** Comparative analysis of control performance metrics.

Index	NN-PID	NTSM	FONTSM	RBF-FONTSM	WRBF-FONTSM
RMSE	3.3073	0.9740	0.4316	0.3226	0.2923
ITAE	142.9545	17.1208	7.7348	6.1303	3.7348
ISU	716.3484	863.5925	700.4151	706.1647	704.5591

Table notes: RMSE:Root Mean Square Error; ITAE:Integral of Time-weighted Absolute Error; ISU: Integral of Squared Control Input.

**Fig 12:**
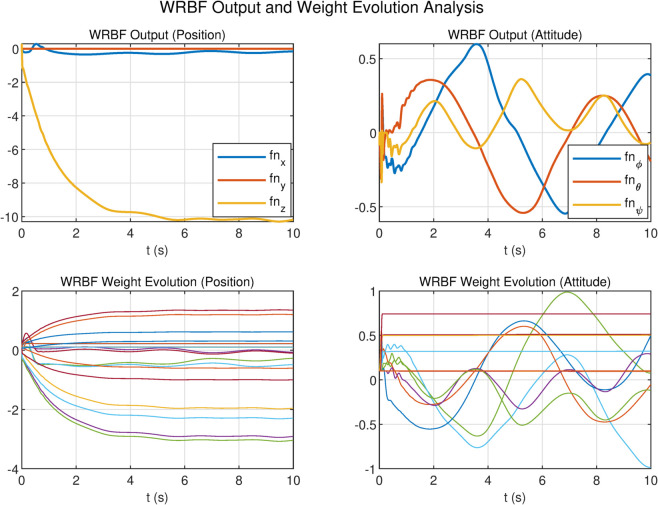
WRBF output and weight evolution analysis.

**Fig 13:**
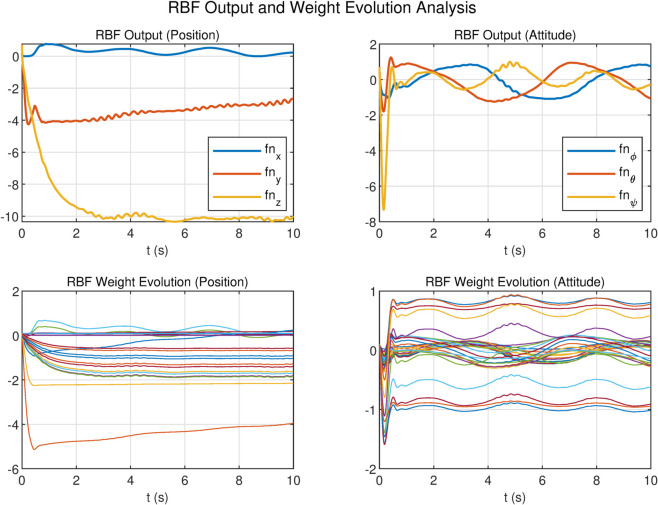
RBF output and weight evolution analysis.

To evaluate the performance advantages of the Wavelet Radial Basis Function (WRBF) over conventional RBF networks, we analyzed output responses and weight evolution in position and attitude channels (Figs 12 and 13), using 10 basis nodes for both networks.

In the position channel, both networks achieved comparable tracking accuracy within [–10,0], though RBF exhibited chattering. WRBF demonstrated superior weight efficiency: its weights spanned 6V (–4V to +2V) versus RBF’s 8V (–6V to +2V), representing a 25% reduction in variation range, indicating more efficient parameter utilization.

The performance gap widened significantly in the attitude channel. RBF output oscillated violently within [–8, 2] with aggressive weight variations in [–2, 1]. In contrast, WRBF suppressed oscillations to [–0.5, 0.5] (90% reduction) and maintained weights in a narrower [–1, 1] range with faster convergence and smoother evolution. These results validate WRBF’s superiority in adaptive control of complex nonlinear systems.

### Computation efficiency analysis

The computational load of the proposed WRBF-FONTSM controller primarily comes from three components: the fractional-order sliding mode control law with O(n) complexity, the WRBF neural network requiring O(M·n) operations, and the adaptive update law with O(M·n) complexity. With parameter configuration (n=6, M=10), the average execution time per control step measured in MATLAB/Simulink (Intel i7) is 0.24 ms, which is well below the typical 5 ms sampling period for UAV systems. This demonstrates sufficient computational margin for real-time implementation, while the linear complexity of all components ensures good scalability for embedded platforms.

## Conclusions

This paper presents a robust and unified 6-DOF control strategy for fully actuated tilt-rotor quadrotors subject to severe model uncertainties and external disturbances. By integrating a WRBF neural network with a fractional-order nonsingular terminal sliding mode controller within a parallel 6-DOF control structure, the proposed method effectively addresses the coupled dynamics, actuator redundancy, and uncertainty compensation challenges inherent to tilt-rotor platforms.

The primary structural innovation involves integrating the WRBF-NN with the FONTSMC under a coordinated control allocation framework. Unlike prior studies limited to joint-space control or static disturbance models, this study redefines the controller architecture to enable real-time trajectory tracking of both position and attitude. The WRBF-NN not only enhances local approximation performance but also builds a dynamic feedback loop for disturbance rejection. Meanwhile, the control allocation module maps the synthesized wrench vector to redundant actuators using the Moore–Penrose pseudoinverse, ensuring optimal use of actuator authority.

These results demonstrate that the WRBF-FONTSMC controller represents not merely a combination of existing techniques, but a coherent framework adapted for complex, over-actuated aerial system. Future work will explore hardware implementation and online optimization of control parameters using This paper presents a robust and unified 6-DOF control strategy for fully actuated tilt-rotor quadrotors subject to severe model uncertainties and external disturbances. By integrating a WRBF neural network with a fractional-order nonsingular terminal sliding mode controller within a parallel 6-DOF control structure, the proposed method effectively addresses the coupled dynamics, actuator redundancy, and uncertainty compensation challenges inherent to tilt-rotor platforms.

The primary structural innovation involves integrating the WRBF-NN with the FONTSMC under a coordinated control allocation framework. Unlike prior studies limited to joint-space control or static disturbance models, this study redefines the controller architecture to enable real-time trajectory tracking of both position and attitude. The WRBF-NN not only enhances local approximation performance but also builds a dynamic feedback loop for disturbance rejection. Meanwhile, the control allocation module maps the synthesized wrench vector to redundant actuators using the Moore–Penrose pseudoinverse, ensuring optimal use of actuator authority.

These results demonstrate that the WRBF-FONTSMC controller represents not merely a combination of existing techniques, but a coherent framework adapted for complex, over-actuated aerial system. Future work will explore hardware implementation and online optimization of control parameters using metaheuristic algorithms.

## Discussion

While the proposed WRBF-FONTSMC controller has demonstrated superior performance in simulations, it is imperative to discuss its associated challenges and limitations to provide a balanced view and guide future research.

1. Computational Complexity and Implementation Feasibility: The enhanced performance comes at the cost of increased computational complexity. The online adaptation of the WRBFNN weights and the numerical evaluation of the Grünwald-Letnikov fractional-order derivative require more computational resources than a standard PID or conventional SMC controller. Although the simulations run efficiently on a desktop PC, real-time implementation on a low-cost, embedded flight controller would require careful optimization. For practical deployment, several optimization strategies are essential. These include adopting fixed-point arithmetic, constraining the memory length in fractional-order calculations, and pruning hidden neurons in the WRBFNN while preserving acceptable performance.
2. Parameter Tuning Burden: The integrated controller introduces a relatively large number of parameters that require tuning, including the FONTSMC parameters (p,q,k,η,β,α), the WRBFNN parameters (centers *c*_*i*_, widths *b*_*i*_, and learning rate γ). While the WRBFNN helps reduce sensitivity to sliding mode gains, identifying a globally optimal parameter set remains challenging. Future work will explore auto-tuning methods, such as meta-heuristic algorithms (e.g., Particle Swarm Optimization), to alleviate this burden and systematically optimize performance.
3. Performance Boundaries and Robustness Limits: The effectiveness of the proposed method relies on the WRBFNN’s ability to learn and compensate for disturbances within its functional approximation bandwidth. The controller may underperform in the face of extremely high-frequency disturbances that fall outside this bandwidth, as the network cannot adapt instantaneously. Furthermore, the control allocation strategy based on the pseudo-inverse does not explicitly handle actuator saturation constraints. In aggressive maneuvers where the computed commands demand actuator outputs beyond their physical limits, performance would degrade without an explicit constrained allocation module, as discussed in Remark 4. Incorporating a quadratic programming-based allocator is a crucial next step for real-world flight tests.

Addressing these limitations provides a clear roadmap for future work, focusing on computational optimization, automatic tuning, and enhanced robustness to actuator constraints and very high-frequency dynamics.
